# Validation study of the Italian version of Temporal Focus Scale: psychometric properties and convergent validity

**DOI:** 10.1186/s40359-020-00510-5

**Published:** 2021-02-01

**Authors:** Pierluigi Diotaiuti, Giuseppe Valente, Stefania Mancone

**Affiliations:** Department of Human Sciences, Society and Health, University of Cassino and Southern Lazio, Campus Folcara, 03043 Cassino, FR Italy

**Keywords:** Temporal focus, Confirmatory analysis, Convergent validity, Satisfaction with life, Anxiety, Depression, Regulatory modes, Self-efficacy

## Abstract

**Background:**

Over the last decade, international research has produced a large number of studies that have stressed the importance of temporal focus in various aspects of the lives of individuals, groups and organizations. This first Italian validation study of the *Temporal Focus Scale* (TFS) has shown a reliable measurement to assess the tendency of individuals to characteristically think about different periods of their lives.

**Methods:**

TFS/I was administered to a sample of 1458 participants, while three other convenience samples (N_1_ = 453; N_2_ = 544; N_3_ = 168) were used for convergent validity testing.

**Results:**

Confirmatory factor analysis confirmed a three-factor solution (including 10 items) with good indices of fit to data, e.g., χ^2^ = 49.533, CFI = 0.992, TLI = 0.986, RMSEA = 0.034, RMSEA 90% CI .018–.048. Convergent validity assessment confirmed predictive indications with variables such as life satisfaction, optimistic/pessimistic orientation, perceived general self-efficacy, self-regulatory modes, anxiety, depression.

**Conclusion:**

The temporal focus has proven to be a significant feature associated with various aspects of both well-being and personal discomfort. By virtue of its good psychometric properties, the TFS can be an integrative tool along with others for a better evaluation of the person’s profile in different contexts such as education, coaching, psychotherapy, counseling and career guidance.

## Background

Despite the unidirectional progression of time, individuals can mentally move back and forth between a fixed past and a more or less vague preview of what awaits them in the future. This premise reinforces the distinction between objective and subjective time. The former refers to the objective passage of time and the latter to the perceived notion of relative time. One inter-individual difference is the different extent to which individuals pay attention to the perceptions of past, present and future [1, 2].

Over the last decade, international research has produced a large number of studies that have shown the importance of temporal focus in various aspects of the lives of individuals, groups and organizations [3–5]. Some researchers have, for example, investigated temporal aspects related to attitudes [6–8], motivations [9–11], individual behaviours [12–14], the effects of time on team processes and performance [15, 16], the role of time in organizational and strategic decisions [17–19] and the dimensions of socio-cultural influence that shape the shared representations of time [20].

The temporal focus is important because it influences the attitudes, decisions and behaviours of the present moment, as highlighted by the research; therefore, it significantly involves aspects of goal setting, individual and collective motivation, quality of performance, learning and self-control of the person.

In 2009 Shipp et al. [21] and colleagues proposed their *Temporal Focus Scale* (TFC). In contrast to earlier scales that conceptualized time perspective as a mix of cognitive, affective, and behavioural influences [22], Shipp et al. [21] focused exclusively on the cognitive component. As such, they define temporal focus as the extent to which individuals devote their attention to the past, present and future. Rather than classify individuals as predominantly one type, this tripartite definition represents three continua in which people can simultaneously be high or low on multiple time frames. People can shift their attention among these time periods, and thinking about one time period does not preclude thinking about the others, and the same individual can have multiple temporal foci.

*TFS* consists of 12 items, including four each for the past, present, and future focus. Responses are assessed on a 7-point scale (1 = never, 3 = sometimes, 5 = frequently; 7 = constantly). For each focus, scale items are averaged to provide an overall score. Differences among the three foci are emphasized, and therefore they should not be combined into an overall temporal focus. As far as the development of the tool is concerned, a domain sampling procedure was used by Shipp et al. [21] to generate items that were consistent with the a priori definition of temporal focus as thinking about the past, present and future. The initial pool consisted of 22 items spread across the three temporal foci, but 12 final items were selected (4 past, 4 present and 4 future). These were subjected to confirmatory factor analysis, and the 3-factor structure was supported.

Convergent validity was assessed through relationships with constructs representing risk-taking, optimism/pessimism, the Big-Five personality factors and job characteristics.

Subsequently, further international studies to evaluate and confirm the factor measurement of TFS included the Irish study of McKay et al. [13], the Japanese study of Chishima et al. [23], and the comparative study of Chishima, MacKay, and Cole [24] carried out with samples of Japanese and British children.

As pointed out by Mohammed and Marhefka [25] in their review of methodological and measurement issues about time perspective, psychometric evidence has been more favourable to *TFS* where they compare this scale with the *ZTPI* (Zimbardo Time Perspective Inventory) [26], *CFCS* (Consideration of Future Consequences Scale) [27], *OFTPS* (Occupational Future Time Perspective Scale) [28], including reliability coefficients, confirmatory factor analyses, and convergent, discriminant, and predictive validity evidence. Furthermore, they claim that, as a relative newcomer to time perspective measurement, the scale could benefit from further research examining its psychometric qualities.

At present, the Italian context does not yet have a homologous instrument to measure temporal focus. Moreover, the time perspective includes only Italian validation studies of short versions of the ZTPI [29, 30]. In order to fill this gap, and following the indication of Worrell et al. [31], who in their review emphasize the problematic nature of a single measure of time perspective (rather than an overarching construct) and therefore the preference for a more robust mode of a separate evaluation of its individual aspects (such as temporal depth, time attitude, consideration of future consequences, possible selves, temporal focus), in this paper we have seen fit to develop and present our preliminary validation study for an Italian version of the *Temporal Focus Scale (TFS-I)*. In order to measure the convergent validity of the scale, we have considered it appropriate to verify the associations with the main constructs already used in previous international studies: anxiety [32–35], depression [32, 36], life satisfaction and subjective well-being [37–41], optimism [21], self-efficacy [42], regulatory modes [43, 44]. In connection with this, we have therefore also hypothesized that the higher the *Current Focus,* the higher the *Optimism, Satisfaction with Life, and Self-Efficacy* would be; (b) the higher the *Past Focus,* the lower the *Satisfaction with Life* and *Self-Efficacy*, while the higher *Depression* and the *Assessment* would be; (c) the higher the *Future Focus,* the higher the *Locomotion* and also the *Anxiety* would be.

## Materials and methods

### Linguistic procedures

The translation of the *TFS* followed forward and backward translations of the original scale, according to the EORTC translation guidelines [45]. Two Italian translators independently completed the forward translation and negotiated any differences in the two versions. The reconciled Italian version was then given to two English translators, who independently back-translated the measure. Any discrepancies were discussed and resolved, and modifications were made in the *TFS* to take into account any rewording to improve the conceptual relevance and comprehension of the items. Finally, a small focus group of 10 components was convened and structured so that three different age groups (20–30; 31–40; 41–50), both genders and subjects with low-medium and high educational qualifications, were represented within it. The discussion held on each item after the administration of the TFS scale did not reveal problems of comprehensibility or literacy discrepancies.

### Participants and administration

As reported in Abbas et al. [46] and Groves and Couper [47] have emphasized that the method of data collection is a significant aspect in order to obtain accurate data or information. For the present study, the sample size planning was based on the ability to verify an adequate fit of *TFS* starting with a translation of the full English version, which included a three-factor model with 12 manifest variables. Using the root-mean-square error of approximation (RMSEA) as the measure of model fit, a minimum of 120 participants provides a 90% power level to test RMSEA ≤ 0.05 when RMSEA = 0.08, using a 0.05 significance level [48]. Participants were recruited through the forwarding of a contact in which the goals as well as function of the study were mentioned. Subjects were invited to enter a specific link found in the same notice, after which they filled in and posted the answers telematically and digitally. Participants were assured anonymity and also the use of information in aggregate type for research purposes. A total of 2500 contact emails were sent. As far as the drop-out ratio is concerned, 88 participants dropped out after beginning to fill it in, therefore 1458 (478 males and 980 females with an average age of 30.97 and SD = 11.59) completed questionnaires were finally collected. The convergent validity was tested using three additional convenient samples of participants, recruited online as well, consisting of a total 1,063 individuals. N_1_ = 453 (155 males), M_age_ 30.62 and SD = 12.46; N_2_ = 544 (150 males), M_age_ 31.59 and SD = 11.61; N_3_ = 168 (66 males), M_age_ 27.14 and SD = 8.60, respectively. In this case, the inclusion criterion was the non-participation in the previous administration. The recruitment phase was carried out in the months of January and February 2020.

### Measures

(a) *Temporal Focus Scale* (TFS) [21]: 12 items articulated into three factors (4 items per factor): *Past Focus*, *Future Focus*, *Current Focus*. Likert scale with an interval of seven points from 1 (never) to 7 (always). (b) *Life Orientation Test* (LOT) [49, 50] consists of four positively-phrased and four negatively-phrased items; participants were asked to indicate the extent to which they agreed on a 4-point scale (i.e., from 1 [strongly disagree] to 4 [strongly agree]) with positively-phrased statements such as “In uncertain times, I usually expect the best” and negatively-phrased statements such as “I rarely count on good things happening to me”. Reliability for this study: Cronbach’s raw alpha (α) [51, 52] = 0.74; McDonald’s omega (ω) [53] = 0.75; [CIs 95% 0.704; 0.777].

(c) *Satisfaction with Life Scale* (SLS) [54, 55]: a 5-item scale designed to measure global cognitive judgments of one’s life satisfaction (not a measure of either positive or negative affect). Participants indicate how much they agree or disagree with each of the 5 items using a 7-point scale that ranges from 7 (strongly agree) to 1 (strongly disagree). Reliability measures for this study: α = 0.85; ω = 0.86; [CIs 95% 0.829; 0.872].

(d) The *Generalised Self-Efficacy Scale* [56, 57] consisted of 10 items on a 4-point Likert scale ranging from 1 (completely false) to 4 intervals (completely true) and was used to assess the general sense of perceived self-efficacy in order to predict coping with daily nuisances as well as adaptation after experiencing all kinds of stressful life events. The scale refers to the personal agency, i.e. the belief that individual actions are responsible for successful results. Reliability for this study: α = 0.87; ω = 0.88; [CIs 95% 0.857; 0.889].

(e) *Beck Depression Inventory—II* [58, 59] is widely used by clinicians in the screening and tracking of depression symptoms and consists of 21 items that are summed up to create a composite score of depression. Examples of these items include questions regarding changes in sleep patterns, difficulty with concentration, sadness, self-dislike, crying, loss of energy, and suicidal thoughts, in which four response options are presented on a scale of 0 to 3. For example, to measure pessimism (item 2) the response options used range from “I am not particularly discouraged about the future” (score of 0) to “The future is hopeless and things cannot improve” (score of 3). These items were designed to capture depression as defined by the *Diagnostic and Statistical Manual of Mental Disorders, Fourth edition* [60]. Reliability for this study: α = 0.90; ω = 0.92; [CIs 95% 0.885; 0.910].

(f) *Endler Multidimensional Anxiety Scale* [61, 62]: the tool distinguishes between state and trait anxiety and assumes that both are multidimensional constructs. The EMAS-State (EMAS-S) is a 20-item measure that assesses state anxiety on a 1–5 point intensity scale. The EMAS-S assesses both cognitive-worry and autonomic-emotional facets of state anxiety (10 items each). Reliability measures for this study: α = 0.92; ω = 0.93; [CIs 95% 0.899; 0.935]. The facets of the EMAS-Trait (EMAS-T) scale are: social evaluation (SE), physical danger (PD), ambiguous (AM), and daily routines (DR). There are 15 items per facet (60 items in total). The EMAS-T is also rated on a 1–5 point intensity scale. Reliability for this study: α = 0.72; ω = 0.76; [CIs 95% 0.641; 0.788].

(g) *Scale of Regulatory Modes* [63, 64] composed of 24 items (12 for the measure of *Assessment Mode* and 12 for the measure of *Locomotion Mode*) 6-point Likert (from 1 = completely disagree to 6 = completely agree). Assessment is the comparative component of the system of regulation of the Self, as a tendency to critically assess the state in which we are in relation to other alternatives in order to achieve the goals in the best possible way. Reliability measures for this study: α = 0.71; ω = 0.71; [CIs 95% 0.631; 0.770]. Locomotion, on the contrary, is the component of our self-adjusting system dedicated to controlling the movement by state and its maintenance to achieve an objective in a simple way and without distractions or delays. Reliability for this study: α = 0.75; ω = 0.76; [CIs 95% 0.691; 0.806].

## Statistical analysis

In order to carry out the statistical analyses we used the package SPSS v. 22 for the verification of the univariate and multivariate hypotheses, for the exploratory factor analysis (EFA) with Maximum Likelihood (ML) and Promax rotation, for the assessment of internal consistency through Cronbach’s raw α coefficient, for the assessment of the significance of correlation coefficients with bootstrap CIs in order to test the convergent validity of the tool; we used the package JASP 0.12.2 to assess McDonald’s ω coefficient; while the Confirmatory Factor Analysis (CFA) used as an extraction method was performed using IBM Amos Graphics 18.

To test the adequacy of the CFA model, as suggested by technical literature [65], Chi-square, CFI (*Comparative Fit Index*), TLI (*Tucker-Lewis Index*) and RMSEA (*Root-Mean-Square Error of Approximation*) were used as relevant fit indicators, with CFI and TLI > 0.95 and RMSEA < 0.06 as excellent model fit indicators [66].

Convergent validity was determined by comparing the correlations between the Temporal Focus Scale factors and the factors that make up LOT-R, SWLS, GSES, BDI-II, EMAS, RMS, by considering, to this end, the constructs already used in previous international studies, as shown in the following Table 1.

**Table 1:** Constructs used in previous temporal focus studies

Constructs	Association evidence	References
Optimism	Past focus was negatively related to optimism	[21]
Satisfaction with life and subjective well-being	Inverse association between past focus and life satisfaction; relationship between a balanced time perspective and subjective well-being	[37–41]
Self-efficacy	Future orientation was associated with higher self-efficacy	[42]
Depression	Depressive rumination was associated with past orientation	[32, 36]
Anxiety	Persons with anxiety symptoms were more prone to look upon their future with worry and negative anticipation and to recall their past with regret and aversive feelings	[32–35]
Regulatory modes	Locomotion was characterized by future focus and reluctance to revisit the past; assessment showed a positive relationship with nostalgia	[43, 44]

As previously stated, the convergent validity assessment was carried out using three additional samples. In the first (N_1_) LOT-R, SWLS were associated in addition to TFS; in the second (N_2_), GSES, BDI-II; in the third (N_3_), EMAS, RMS. This partition was motivated by the desire to avoid a possible fatigue effect with a single extended administration. It has been hypothesized from these analyses that (a) the higher the *Current Focus,* the higher the *Optimism, Satisfaction with Life, and Self-Efficacy* would be; (b) the higher the *Past Focus,* the lower the *Satisfaction with Life* and *Self-Efficacy*, while the higher *Depression* and the *Assessment* would be; (c) the higher the *Future Focus,* the higher the *Locomotion* and also the *Anxiety* would be been. In order to measure convergent validity, Pearson coefficients with their CIs were computed.

## Results

The verification of the assumptions of univariate and multivariate normality has been conducted using the procedure for the standardization of the variables, erasing the outlier cases with values greater than 3; secondly, after calculating the Mahlanobis Distance, eliminating the multivariate outlier cases with D^2^ greater than the critical value, calculated by considering chi-square as the reference distribution (level *p* < 0.001) with *p* degrees of liberty equal to the number of variables [67]. The calculation of the Mardia Index (average of the squares of the Malhanobis Distances) produced a coefficient (180.46) lower than the limit value (195). This selection of cases from the original matrix implied the elimination of 168 participants, whose high values of the outliers made us assume that the compilation was not very accurate and reliable, also considering that the administration was done in telematic mode and not in the presence of the operators. Therefore, the rest of the validation procedure was carried out with 1458 cases, 478 of which were males (32.8%) and 980 females (67.2%). The average age was 30.97 with SD = 11.59.

The evaluation of the metric properties of the scale was conducted through a confirming analysis (CFA) designed to test the goodness of the three-dimensional model adopted by Shipp et al. [21]. The averages and standard deviations for the single items are reported in the following Table 2.

**Table 2:** Descriptive statistics of the Italian Temporal Focus Scale (TFS-I) (N = 1458)

Item	M	SD	Bootstrap CI 95%
Item 1	3.70	.99	(3.63–3.77)
Item 2	5.43	1.28	(5.34–5.51)
Item 3	4.69	1.39	(4.59–4.78)
Item 4	5.25	1.23	(5.15–5.34)
Item 5	4.62	1.43	(4.52–4.72)
Item 6	3.67	1.02	(3.59–3.74)
Item 7	4.38	1.33	(4.28–4.47)
Item 8	4.66	1.26	(4.56–4.75)
Item 9	3.92	1.03	(3.85–4.00)
Item 10	4.49	1.28	(4.40–4.58)
Item 11	3.51	.97	(3.44–3.59)
Item 12	4.43	1.34	(4.34–4.53)

*M* mean, *SD* standard deviation, *CI* confidence interval

In order to examine the validity of a 12-item construct, a confirmatory factor analysis was performed. The results obtained by considering three factors and 12 items did not show a good fit to the data. Therefore, the existence of a lower number of items was verified by performing an EFA with ML, therefore items 5 and 10 were removed because they were found to damage the fit between the model and the covariance structure. Through the omission of these two items the following fit values were reached: Kaiser–Meyer–Olkin (KMO) index score was 0.752, Chi-squared Test < 0.01; RMSEA = 0.058; RMSEA 90% 0.43–0.074; TLI = 0.96.

Table 3 shows the model matrix with saturations on the three identified factors, McDonald’s ω and Crombach’s Alpha values, Guttman Split-Half Coefficients, Corrected item/total correlations, factor intercorrelations; while in Table 4 the factorial interrelationships are reported.

**Table 3:** Pattern matrix EFA (10 items)

	Past focus	Future focus	Current focus
Item 6	.877	− .031	− .033
Item 1	.771	− .044	.009
Item 11	.728	.043	− .013
Item 9	.625	.039	.046
Item 7	− .003	.831	− .040
Item 12	− .011	.816	.000
Item 3	.018	.757	.042
Item 8	− .008	− .047	.751
Item 4	.060	.087	.704
Item 2	− .042	− .037	.672
*α*	.84	.84	.75
ω	.84	.84	.75
*λ6*	.80	.78	.67
r*	.56	.64	.50

Extraction Method: Maximum Likelihood. Rotation Method: Promax with Kaiser Normalization. Rotation converged in 4 iterations. Item number relates to Shipp et al. 2009. *α* = Cronbach’s alpha; ω = McDonald’s omega;* λ6* = Gutmann’s lamda; r* = average inter-item correlation

**Table 4:** Factor inter-correlations

	Past focus	Future focus
Future focus	0.225	
Current focus	− 0.269	0.103

**Fig. 1:**
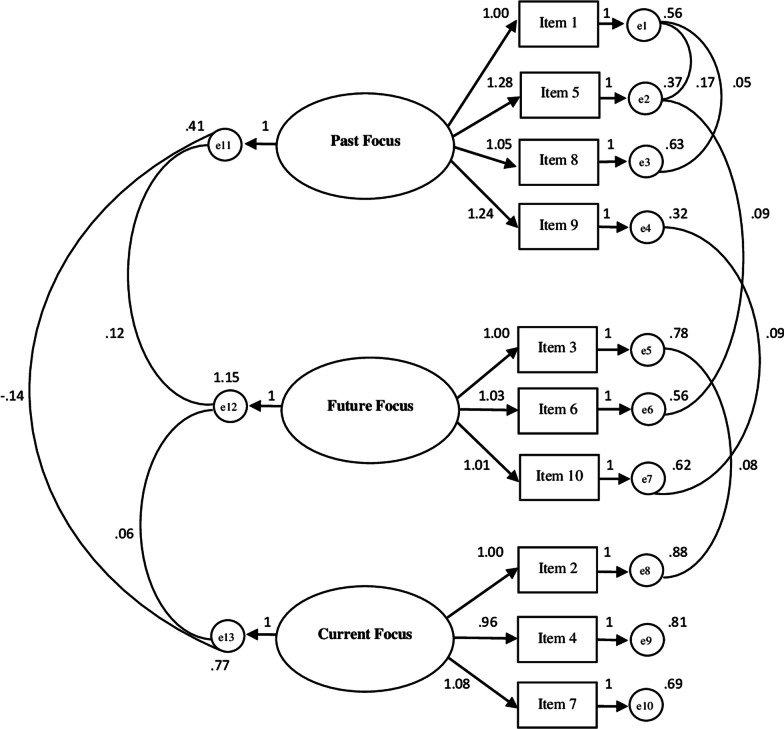
Path diagram of the confirmatory analysis concerning TFS-I (10 items)

The confirmatory factor analysis (see Fig. 1) confirmed that the model with three related factors and 10 items presented overall good indices of fit to data: Chi-square = 49.533; CFI = 0.992; TLI = 0.986; RMSEA = 0.034 and RMSEA 90% CI [0.018–0.048]. The first factor measures *Past Focus* (4 items); the second factor measures *Future Focus* (3 items); the third factor measures *Current Focus* (3 items).

Three other samples were used for convergent validity testing: correlations with LOT-r and SWLS were performed by administering a sample of 453 individuals (155 males and 298 females) with an average age of 30.62 and SD = 12.46; correlations with GSE and BDI-II were carried out by administering a sample of 544 individuals (150 males and 394 females) with an average age of 31.59 and SD = 11.61; correlations with EMAS and RMS were performed by administering a sample of 168 individuals (66 males and 102 females) with an average age of 27.14 and SD = 8.60. In relation to the hypotheses stated with regard to these associations, as shown in the following Tables 5, 6 and 7, the results obtained have substantially confirmed the directions assumed.

**Table 5:** Bivariate correlations between TFS/I, BDI-II and GSE

N = 544	M (SD)	SKE (SE)	KUR (SE)	PA	CU	FU	DTOT	DSOM	DCOG	GSE
*TFS*										
PA	3.91 (1.20)	.611 (.105)	− .114 (.209)	1						
CU	4.89 (1.13)	− .158 (.105)	− .122 (.209)	− .170^**^	1					
				[− .09, − .25]						
FU	4.47 (1.28)	.099 (.105)	− .455 (.209)	.218^**^	.142^**^	1				
				[.30, .14]	[.22, .06]					
*BDI-II*										
DTOT	32.86 (10.3)	1.25 (.105)	2.15 (.209)	.454^**^	− .322^**^	.072	1			
				[.52, .38]	[− .24, − .40]	[.15, − .01]				
DSOM	20.24 (6.81)	1.46 (.105)	1.80 (.209)	.424^**^	− .294^**^	.063	.962^**^	1		
				[.49, .35]	[− .21, − .37]	[.15, − .02]	[.97, .95]			
DCOG	12.62 (4.22)	1.63 (.105)	2. 24 (.209)	.429^**^	− .315^**^	.074	.898^**^	.745^**^	1	
				[.49, .36]	[− .24, − .39]	[.16, − .01]	[.91, .88]	[.78, .70]		
*GSE*										
	3.75 (.653)	− .567 (.105)	.984 (.209)	− .105^*^	.244^**^	.058	− .324^**^	− .266^**^	− .363^**^	1
				[− .02, − .19]	[.32, .16]	[.14, − .03]	[− .25, − .40]	[− .19, − .34]	[− .29, − .43]	

*TFS* temporal focus scale, *PA* past focus, *CU* current focus, *FU* future focus, *BDI-II* beck depression inventory-II, *DEPTOT* total depression, *DSOM* somatic depression, *DCOG* cognitive depression, *GSE* generalized self-efficacy scale

**Correlation is significant at the 0.01 level (2-tailed)

*Correlation is significant at the 0.05 level (2-tailed). For BDI-II Spearman’s correlation has been used. Values in square brackets indicate the 95% confidence interval for each correlation

**Table 6:** Bivariate correlations between TFS/I, LOT-R and SWLS

N = 453	M (SD)	SKE (SE)	KUR (SE)	PA	CU	FU	LOT-R	SWLS
*TFS*								
PA	3.92 (1.09)	.884 (.115)	.161 (.229)	1				
CU	5.17 (1.09)	− .303 (.115)	− .182 (.229)	− .135^**^	1			
				[.04, − .22]				
FU	4.50 (1.28)	.039 (.115)	− .557 (.229)	.238^**^	.073	1		
				[.32, .15]	[.16, − .02]			
*LOT-R*								
LOT-R	20.96 (4.38)	.619 (.115)	.432 (.229)	− .094^*^	.282^**^	− .003	1	
				[− .01, − .18]	[.36, .19]	[.09, − .09]		
*SWLS*								
SWLS	4.65 (1.31)	− .350 (.115)	− .635 (.229)	− .175^**^	.345^**^	− .071	.345^**^	1
				[− .08, − .26]	[.42, .26]	[.02, − .16]	[.42, .26]	

*TFS* temporal focus scale, *PA* past focus, *CU* current focus, *FU* future focus, *LOT-R* life orientation test, *SWLS* satisfaction with life scale

**Correlation is significant at the 0.01 level (2-tailed)

*Correlation is significant at the 0.05 level (2-tailed). For BDI-II and LOT-R Spearman’s correlation has been used. Values in square brackets indicate the 95% confidence interval for each correlation

**Table 7:** Bivariate correlations between TFS/I, LOT-R and SWLS

N = 168	M (SD)	SKE (SE)	KUR (SE)	PA	CU	FU	EMAS-S	EMAS-T	LOC	ASS
*TFS*										
PA	3.48 (1.07)	.673 (.187)	.919 (.373)	1						
CU	5.44 (1.11)	− .107 (.187)	− .839 (.373)	− .124	1					
				[.03, − .27]						
FU	4.52 (1.39)	.120 (.187)	− .701 (.373)	.366^**^	.287^**^	1				
				[.49, .23]	[.42, .14]					
*EMAS*										
EMAS-S	1.42 (.461)	1.06 (.187)	− .161 (.373)	.080	− .084	.273^**^	1			
				[.23, − .07]	[.07, − .23]	[.41, .13]				
EMAS-T	3.84 (.541)	− .095 (.187)	− .533 (.373)	.124	.291^**^	.325^**^	.061	1		
				[.27, − .03]	[.42, .15]	[.45, .18]	[.21, − .09]			
*RMS*										
LOC	4.82 (.544)	− .529 (.187)	.003 (.373)	.204^**^	.206^**^	.023	− .249^**^	.180^*^	1	
				[.34, .05]	[.35, .06]	[.17, − .13]	[− .10, − .39]	[.32, .03]		
ASS	3.31 (.584)	− .451 (.187)	− .302 (.373)	.190^*^	− .033	.093	.227^**^	.324^**^	− .048	1
				[.33, .04]	[.12, − .18]	[.24, − .06]	[.37, .08]	[.45, .18]	[.10, − .20]	

*TFS* temporal focus scale, *PA* past focus, *CU* current focus, *FU* future focus, *EMAS* endler multidimensional anxiety scale, *EMAS-S* state anxiety, *EMAS-T* trait anxiety, *RMS* regulatory modes scale, *LOC* locomotion, *ASS* assessment

**Correlation is significant at the 0.01 level (2-tailed)

*Correlation is significant at the 0.05 level (2-tailed). For BDI-II and LOT-R Spearman’s correlation has been used. Values in square brackets indicate the 95% confidence interval for each correlation

The strength of the associations appeared to be substantially in line with other studies. A difference can be found in the values of association with the Assessment and Locomotion subscales. In the first case, the measure of association between Assessment and temporal orientation to the Past was less strong (0.19*) compared to what was highlighted in Choy and Cheung (0.63**) [44]. Whereas, although Locomotion presented associations with both Past and Present, it has not recorded significant association with the Future temporal orientation, as highlighted by Choy and Cheung in this case as well (0.71**) [44].

Overall the results have however confirmed the assumed directions of correlation; moreover, the measure proved good convergent validity with the scales considered and consequently its usefulness in describing the dominant temporal focus of the person and indirectly providing indications about his psychological well-being as well.

In Table 8 the internal reliability of the three samples are shown comparatively with their confidence intervals. McDonald’s ω and Alpha coefficients for these convergent administrations ranged from 0.78 to 0.88 (*Past Focus*), from 0.80 to 0.83 (*Future Focus*), from 70. to 0.72 (*Current Focus*), respectively.

**Table 8:** Internal reliabilities of the three samples

	Sample 1 (N = 544)			Sample 2 (N = 453)			Sample 3 (N = 168)		
	*α*	ω	C.I	*α*	ω	C.I	*α*	ω	C.I
Past focus	.88	.88	[.86, .89]	.87	.87	[.85, .89]	.78	.80	[.72, .83]
Current focus	.71	.72	[.67, .75]	.70	.71	[.65, .75]	.70	.71	[.61, .77]
Future focus	.82	.82	[.80, .85]	.83	.83	[.80, .86]	.80	.80	[.74, .85]

α = Cronbach’s alpha; ω = McDonald’s omega; C.I. = 95% Confidence Interval

The following Table 9 reports the English and Italian versions of the *TFS-I*, and the grouping of the items on respective factors.

**Table 9:** Temporal focus scale (TFS-I)

English version	Italian version
1. I think about things from my past (PF)	1. Penso a cose vissute nel mio passato
2. I live my life in the present (CF)	2. Vivo la mia vita nel presente
3. I think about what my future has in store (FF)	3. Penso a ciò che ha in serbo il mio futuro
4. I focus on what is currently happening in my life (CF)	4. Mi concentro su ciò che accade attualmente nella mia vita
5. I replay memories of the past in my mind (PF)	5. Rivivo nella mia mente i ricordi del passato
6. I imagine what tomorrow will bring for me (FF)	6. Immagino cosa mi porterà il domani
7. My mind is on the here and now (CF)	7. La mia mente è indirizzata sul qui e ora
8. I reflect on what has happened in my life (PF)	8. Rifletto su ciò che è accaduto nella mia vita
9. I think back to my earlier days (PF)	9. Ripenso ai giorni passati
10. I think about times to come (FF)	10. Penso ai momenti che verranno

*PF* past focus, *FF* future focus, *CF* current focus

As far as the scoring of the instrument is concerned, the 10 items in total are distributed over three factors: the first comprises 4 items, while the second and third comprise three items each. Every item has a scoring range from 1 (never) to 7 (always). The person is asked to indicate how often they direct attention to the listed thoughts. The scoring calculation produces, through a summation of the scores of the component items, separate measurements for each factor. Therefore, *Past Focus*: 1 + 5 + 8 + 9; *Future Focus*: 3 + 6 + 10; *Current Focus*: 2 + 4 + 7. The first factor can have a total score range from 4 to 28, while the second and third factors can have a total score range from 3 to 21. Based on the distribution of the scores obtained from the normative sample, the cut-off criteria, differentiated by gender, have been identified and reported in the following Table 10.

**Table 10:** Scoring directions of TFS-I

Factor	Low	Medium	High	*M*	DS	SE	SK	SE	KU	SE
Total sample (N = 1458)										
PF	4–11	12–16	17–28	14.80	3.28	.12	.54	.09	− .56	.18
FF	3–10	11–15	16–21	13.50	3.54	.13	.28	.09	− .61	.18
CF	3–12	13–16	17–21	15.33	3.08	.11	− .07	.09	-.52	.18
Males (N = 478)										
PF	4–11	12–15	16–28	14.32	3.33	.22	.65	.16	− .24	.31
FF	3–9	10–14	15–21	12.87	3.62	.23	.48	.16	− .39	.31
CF	3–12	13–16	17–21	15.19	3.15	.20	.10	.16	− .61	.31
Females (N = 980)										
PF	4–11	12–17	18–28	15.03	3.24	.15	.51	.11	− .69	.22
FF	3–10	11–15	16–21	13.81	3.46	.16	.21	.11	− .65	.22
CF	3–12	13–16	17–21	15.40	3.05	.14	− .16	.11	− .44	.22

The score ranges (low, medium, high) correspond to the percentiles below 25, 25–75, above 75

*PF* past focus, *FF* future focus, *CF* current focus, *SE* standard error, *SK* skeweness, *KU* kurtosis

## Discussion

The aim of this work was to develop and present a preliminary validation study for an Italian version of the *Temporal Focus Scale (TFS-I).* The analyses carried out led to the definition of a scale composed of a total of 10 items that converge separately on three factors. The first factor measures the person’s propensity to focus attention on events in their past. The convergent validity analysis indicated the significant association with the two components (somatic-affective and cognitive) of the depression scale. It can therefore be said that the person with a high score on the Past Focus scale could present mood declines, dysphoria, general dissatisfaction with the present condition of life and the results achieved, melancholy and nostalgia for past events, sadness, pessimism, low self-esteem, propensity to self-criticism, loss of energy and motivation, difficulty in concentration, fatigue and sleep disorders. Excessive and persistent focus on the past can represent a real block for the person, who cannot functionally channel the energies to deal with and solve the tasks of his present condition.

Several studies have pointed out the association of depressive remorse with less capacity to act and less effort and less confidence in one’s own ability to solve problems [32, 68].

Mckay et al. [69], through a study with cluster analysis on a sample of students, reported the highest frequency of psychiatric symptomatology in participants belonging to the cluster with prevalent focus in the past. Perry et al. [37] have shown a positive association between past orientation and low self-esteem in adolescents. Vannikov-Lugassi and Soffer-Dudek [38] reported the positive predictive association of thinking about the past with dissociation mechanisms: depersonalization (DEP), derealization (DER), and absorption (ABS). Other authors have reported the inverse association between past focus and life satisfaction [39, 40]. This direction in correlation has emerged in our study as well.

The second factor of TFS measures the propensity of the person to think about the future. The convergent validity analysis reported positive correlations with measures of anxiety (state and trait). Therefore, the anxious individual lives in constant fear that the uncertainty of the present will lead to a near future characterized by negative outcomes that would find him/her incapable of managing the situation. In an attempt to control the anxiety, he/she anticipates a future that does not exist but is lived in an excessively threatening way. Some scholars have investigated associations between time perspective and trait anxiety among college students [22] and between time perspective and anxiety symptoms [33, 34]. These studies showed that anxiety is associated with Past Negative as well as Future Negative.

Zaleski [70] introduced the concept of Future Anxiety (FA) and regarded it as a personality characteristic where a negative future time perspective precedes the development of anxiety. These findings suggest that anxiety is associated with a predominantly future-oriented time perspective. The results of our study were in line with this research orientation. Altan-Atalay et al. [39] also reported that the interaction of future time perspective with negative urgency was associated with anxiety, indicating that tending to focus on the events that are likely to take place in the future is associated with elevated levels of anxiety in individuals with high levels of negative urgency, that is the tendency to act rashly when you are in trouble.

Finan et al. [40] recently showed the relationship between a clearly past and future-oriented time perspective and anxiety in adolescents, particularly in females. These results highlight the temporal qualities of anxiety and provide support for time perspective as a potential factor for understanding and supporting adolescents with anxiety.

The third factor measures the tendency to focus attention and thoughts on the present moment. In accordance with the literature [21, 71, 72], our results showed a positive association with life satisfaction and optimistic orientation, an inverse association with depressive components, and also a positive association with trait anxiety. For this last result, it is worth pointing out that the tension of the anxious person is usually not only projected into the future but characterizes the attitude and expectations of negative outcome also for the present moments of the person. Several studies have reported that an excess of focus on the present was associated with behaviours that tend to be more risk-seeking [72], aggressive [73], engaged in drug and alcohol use [74], apting to procrastinate in response to situations rather than acting proactively [75].

The research on measurement of temporal focus is currently oriented to identify different approaches to calculate to what extent individuals differ in their attention to the their present, past or future, and therefore define a balance profile of the person and consider measures of distancing from this profile as antecedents of emotional distress and distortion in cognitive evaluation [76–79]. Therefore, temporal focus may act as a profile that can be balanced or unbalanced [80], and a stronger balance among the past, present and future seems to lead to a greater well-being [81–83].

Although our validation contribution also provided a key for the evaluation of the scores, (classified as low, medium or high using a percentile distribution < 25% and > 75%), there was the limitation that we have not yet identified for TFS appropriate criteria to define the profile of the person, with respect to this dimension of time, as balanced or unbalanced through a possible analysis of clusters, in accordance with the directions of the most recent literature mentioned above, which has produced indications referring to the ZTPI. A further limitation was the lack of an in-depth measurement of the invariance of the scale with respect to the age cohorts of the participants, as suggested by the study by Irish et al. [83]. In addition, the absence from the procedural plan of a longitudinal administration to test temporal stability and predictive validity of TFS was a current limitation of the work. The results related to the associations which emerged with anxiety and depression would need further investigation through the use of clinical samples. These could then be the possible cues for further continuation of the work.

At any rate, the main significance of this study consists in the attempt to fill a gap in the Italian psychometric context which, despite significant interest in the subject demonstrated by the abundance of international scientific studies in the last 10 years, did not have a specific instrument to measure temporal focus, whereas in reference to Time Perspective there were only validation studies concerning short versions of the ZTPI.

## Conclusion

The first Italian validation study of the Temporal Focus Scale has demonstrated that this version is a reliable measurement to assess the tendency of individuals to think about the past, present and future. The convergent validity assessment confirmed predictive indications with variables such as life satisfaction, optimistic/pessimistic orientation, perceived general self-efficacy, self-regulatory modes, anxiety and depression. Temporal focus has proven to be a significant feature associated with various aspects of both personal well-being and distress. Due to its good psychometric properties, TFS can be a supplementary tool, among others, for a more accurate assessment of a person’s profile in different contexts, such as education, coaching, psychotherapy, counseling and career guidance. The availability of this new tool is also intended to be a stimulus to promote new comparative studies in Italy in order to test the adequacy of the temporal focus model on specific samples of the population (and clinical population), and investigate the differences between individuals and internal changes that can occur due to aging, significant life events, or in the light of an individual’s status.

## Data Availability

The datasets during and/or analysed during the current study are available from the corresponding author on reasonable request.
